# Biodeterioration of canvas paintings: microbial role and development of sustainable treatments for biocontrol

**DOI:** 10.1007/s00253-025-13553-8

**Published:** 2025-08-13

**Authors:** Giovanna Climaco, Gianmaria Oliva, Paola Fiore, Consiglia Tedesco, Stefano Castiglione, Giovanni Vigliotta

**Affiliations:** 1 Department of Design and Applied Arts, Conservation School, Fine Arts Academy of Naples, 80138 Naples, NA Italy; 2 Department of Chemistry and Biology “A. Zambelli”, University of Salerno, 84084 Fisciano, SA Italy

**Keywords:** Conservation, Cultural heritage, Artwork, Biodegradation, Plant extracts, Disinfection

## Abstract

**Abstract:**

Biodeterioration of paintings, caused by microorganisms interacting with the organic/inorganic compounds of the canvas, represents a serious problem for preserving cultural heritage. In our study, the microbial degradation caused on an eighteenth century painting “Sant’Anna, San Gioacchino e la Vergine Bambina” was investigated. Seventeen bacterial and six fungal strains on the altered parts of the canvas were identified, and their deteriorating ability were evaluated on two pictorial pigments: the yellow ochre and the ivory black. We recognized that microorganisms interacted with these pigments and modified their chromatic features. Furthermore, we adopted an eco-friendly antimicrobial treatment based on natural plant extracts (thymus, rosemary, and garlic) as an alternative to conventional biocides and highlighted how rosemary (*Salvia rosmarinus*) extract was the best and inhibited 74% of the isolated bacterial strains. When the extract was applied on the contaminated canvas, it reduced bacterial colonization by ~ 75% in only 48 h and eliminated the fungi within 7 days. The extract application was optimized, to minimize potential alterations of the painting caused by the extract, by adopting different strategies: (i) direct nebulization, (ii) Evolon® tissue (an innovative technical polyester-polyamide textile), and (iii) pretreatment with cyclomethicone D5. Finally, we also verified that it did not cause chromatic variations on the canvas confirming its suitability for conservation purposes. Our study provides new insights on the role of microorganisms in the deterioration of cultural heritage and highlights the potentiality of plant-based antimicrobials as sustainable, non-invasive, and alternative to traditional methods for the artwork preservation. Future research should focus on long-term efficacy assessments and formulation optimization to enhance applicability in heritage conservation practices.

**Key points:**

• *Identification of 17 bacteria and 6 fungi from a deteriorate canvas painting*

• *Microorganisms altered yellow ochre and ivory black, causing chromatic changes*

• *Rosemary extract applied on canvas reduced microbial colonization*

**Supplementary Information:**

The online version contains supplementary material available at 10.1007/s00253-025-13553-8.

## Introduction

Biodeterioration of paint pigments on canvas represents a significant challenge for the conservation of this kind of cultural heritage. An important role in biodeterioration is attributable to the colonization of microorganisms (fungi and bacteria) and their interactions with organic/inorganic compounds present in the canvas painting (Joseph 2021). Organic materials in paintings can be found in the cellulose support and in the binders, that can be made by oils, resins, egg yolk, or animal glue. The latter are also used during conservation treatments (Ciferri 1999; Poyatos et al. 2018). Pictorial pigments, on the other hand, are generally characterized by inorganic compounds, such as metal oxides, carbonates, sulphates, as well as heavy metals (Santos et al. 2009). The presence of organic and inorganic compounds represents a nutrient-rich environment that favors the colonization from microorganisms, especially on the back side of the canvas, where the microclimate between the painting and the wall can further contribute to their development (Rubio and Bolívar 1997; Capodicasa et al. 2010). In addition, a poor state of conservation can favor microbial attack. In fact, heat and humidity can favor microbial growth and accelerate the deterioration of the paintings. In the long term, these interactions lead to visible degradation effects, including chromatic alteration and structural deterioration of the paint.

Some of the microorganisms responsible for this deterioration have been identified by several studies. The most common recognized fungi belong to the genera: *Aspergillus*, *Penicillium*, *Cladosporium*, and *Alternaria*, while bacteria belong to the genera: *Microbacterium*, *Staphylococcus*, *Sporosarcina*, *Bacillus*, and *Acinetobacter* (Poyatos et al. 2018). It has been shown that these bacteria produce metabolites, such as organic acids and enzymes which attack the binders and pigments, changing their chemical composition (Di Carlo et al. 2022). Furthermore, they can cause color changes due to oxidation or reduction processes of metal components constituting the pigments (Kujović et al. 2024).

Metabolic activities of deteriorating fungi are still little known; however, it has been observed that they can produce either melanins or other substances that darken the surfaces and penetrate the pictorial layers, thanks to their hyphae, damaging them and causing the loss of paint cohesion (Mendes et al. 2022). These processes, if not counteracted, can compromise the aesthetic and material stability of the paintings (Camuffo 2019). In this regard, disinfection of canvases is essential for treating and preventing the degradation of artworks. However, disinfection techniques must be chosen with extreme care to avoid damaging of supports or the pictorial surfaces. The most common practice uses commercial products with antimicrobial activity (i.e., Rocima 103, Biotin, ProClin 950, Vancide 51, etc.) (Franco-Castillo et al. 2021). Although these are very effective against a wide range of microorganisms, their toxicity remains the major problem for the health of the operator and for the environment, making their use in conservation interventions not too much sustainable (Barresi et al. 2017).

In recent years, scientific research has posed attention to the development of environmentally friendly solutions to (i) limit the damage caused by microorganisms on cultural heritage; (ii) minimize environmental impact; and (iii) ensure the conservation of the artworks. These solutions focused in particular on natural biocides such as secondary metabolites obtained from plants (i.e., essential oils) (Sparacello et al. 2021; Russo and Palla 2023), but also nanoparticles (Kolman et al. 2018), offering safer, cheaper, and sustainable solutions.

Consolidated chemical compounds for the disinfection of cultural heritage, as substitutes for traditional biocides, are certainly essential oils or nanomaterials; in fact, it has widely demonstrated their antimicrobial activity by many scientific publications (Böhme et al. 2020; Baglioni et al. 2021; Spirescu et al. 2021; Puvača et al. 2021; Angane et al. 2022; Maťátková et al. 2022; Carrapiço et al. 2023). However, the applications of these compounds on artworks have several limitations. In the case of essential oils, the volatility and insolubility in water make their application quite complex, and also, can cause undesired optical effects on the artefacts, such as color variations or halos, especially on porous surfaces (i.e., canvas) (Russo and Palla 2023). While for nanoparticles, the limitations are mainly due to darkening or whitening (Oriola-Folch et al. 2020).

The effectiveness of the natural extracts in hydroalcoholic formulation is well known from the scientific literature, and their antimicrobial activity has been widely demonstrated and attributable to the presence of bioactive substances such as polyphenols and flavonoids (Efenberger-Szmechtyk et al. 2021; Stan et al. 2021). In this regard, the effectiveness of the garlic hydroalcoholic extract against numerous microorganisms isolated from cultural heritage with biodeterioration abilities is demonstrated (Corbu et al. 2021). Likewise, it was recognized a good and wide spectrum antimicrobial activity of rosemary hydroalcoholic extract against deteriogenic microbial strains. It was observed that rosemary inhibited the adherence capacity to the inert substrate of *Penicillium chrysogenum* strains isolated from wooden objects or textiles, and the growth of *Bacillus thuringiensis* strains (Corbu et al. 2022).

However, the application of natural extract with water-based products requires long drying times, and this can be a problem since prolonged contact with water promotes the swelling or shrinkage of the canvases and base layers.

**Fig. 1:**
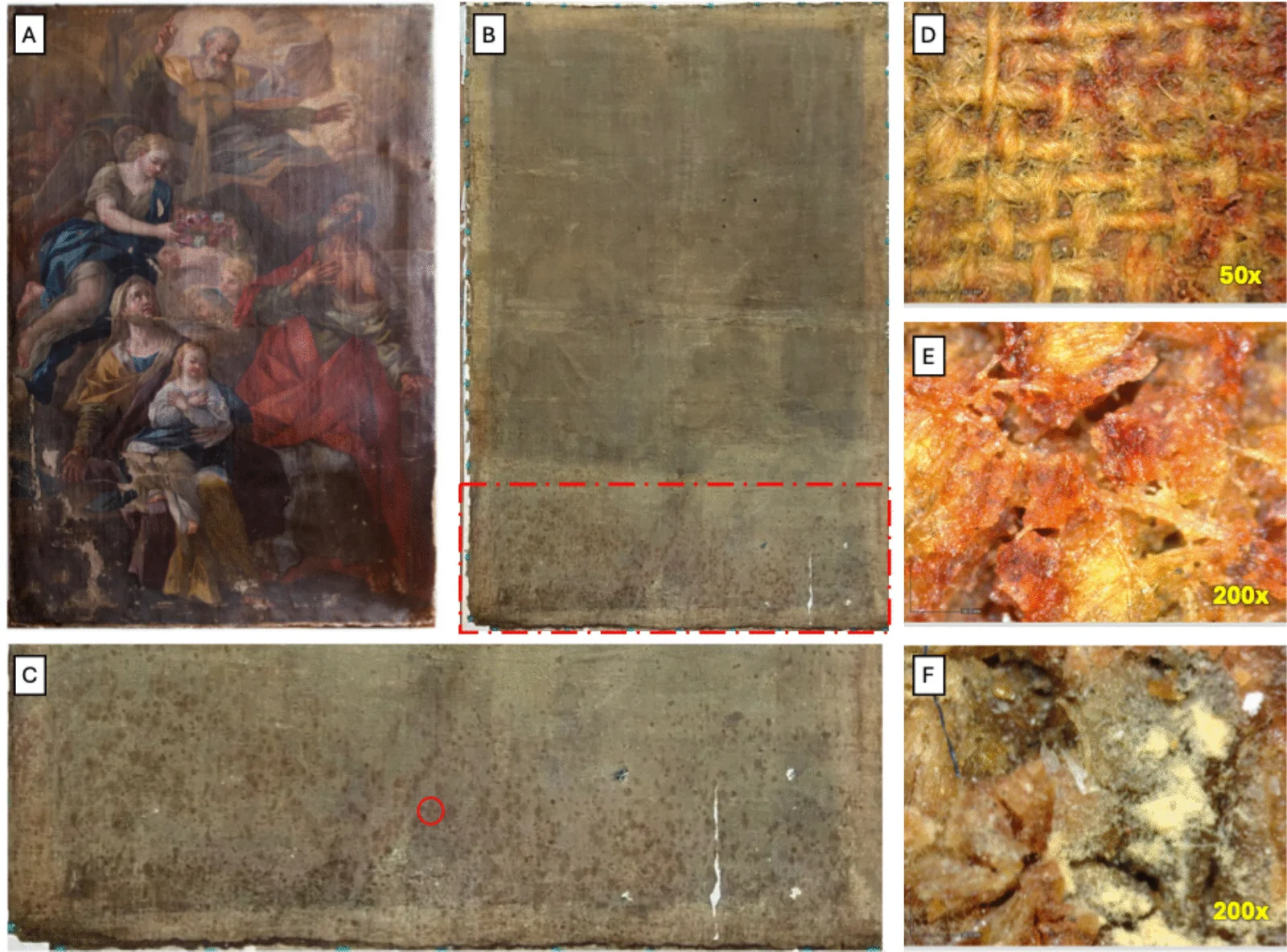
**A** Paint by D. Carlo Malinconico, “Sant’Anna, San Gioacchino e la Vergine bambina”, 1728, oil on canvas. **B**–**C** Back of the canvas. The red rectangle indicated different purple-red-white spots attributable to microbial activity, while red circle indicated the area magnified under microscope in the following panels. **D**-**E**-**F** Magnification of purpler-red-white spots

The present work was conducted on an eighteenth century painting preserved in the Gallery of Naples Academy, entitled “Sant’Anna, San Gioacchino e la Vergine bambina” (Fig. 1) and signed by the artist D. Carlo Malinconico (1705–1784). The painting showed different purple-red-white spots on the lower part of the back of the canvas (Fig. 1B–C) attributable to a possible biodeterioration phenomenon. The aim of this research was to characterize the microbial community colonizing it and evaluate the biodeterioration action of the individual strains through an in vitro approach of their interaction with two pictorial pigments: the yellow ochre and the ivory black. The pigments were chosen based on the stratigraphic analyses carried out on the painting, reviling an abundant presence of ochre and carbonaceous black pigments in the lower part of the artifact, specifically in correspondence of the biological contamination. In parallel, our study also explored the search of a sustainable, effective, and low-cost natural antimicrobial, as an alternative to common disinfection methods, but easily appliable in artifact conservation processes without altering the optical and mechanical properties of the canvas and paint layers.

## Materials and methods

### Textile fiber analysis of relining and original canvas

The back of the original canvas has been covered in the beginning of the twentieth century by a relining canvas that was removed during conservation operations realized at the same time of this study. Also in the case of the relining canvas, it was observed the presence of several red–purple-white spots in correspondence of the same area of original canvas (Supplemental Fig. S1).

To carry out the recognition of textile fibers, two samples of threads were taken from the original canvas (TO1, TO2) and two from the relining canvas (TR1, TR2), to acquiring information on the nature of the yarn, both for the weft and warp threads. Before being positioned on the slide, the individual samples were subjected to two washes: the first carried out with a mixture of water and ethanol (ratio 1:1) and the second with a 1 M HCl solution to remove foreign substances, as well as to allow the fibers to open more easily, facilitating observation under the microscope. The samples were then observed at magnifications of 100 × and 400 ×.

### Ultraviolet fluorescence photography

The UV fluorescence analysis allows to observe the original and non-original added layers directly on the surface of the painting, starting from the most external ones, such as varnishes or protective films, up to the recognition of some pigments and binders, and the distinction of adhesives and products used in the conservation field, based on the color of the response. Furthermore, since the fluorescence of many of these materials tends to increase in intensity with aging, it is possible to carry out a preliminary study on the conservation history of the painting.

In particular, ultraviolet fluorescence photography aims at detecting the visible response generated by irradiation in the UV range 300–400 nm. The fluorescence response was recorded using a camera equipped with special filters (such as the yellow filter—UV cat), allowing the passage of only the visible radiation emitted by the object due to fluorescence, while preventing the reflected UV from being recorded.

### Collection of biological samples

**Fig. 2:**
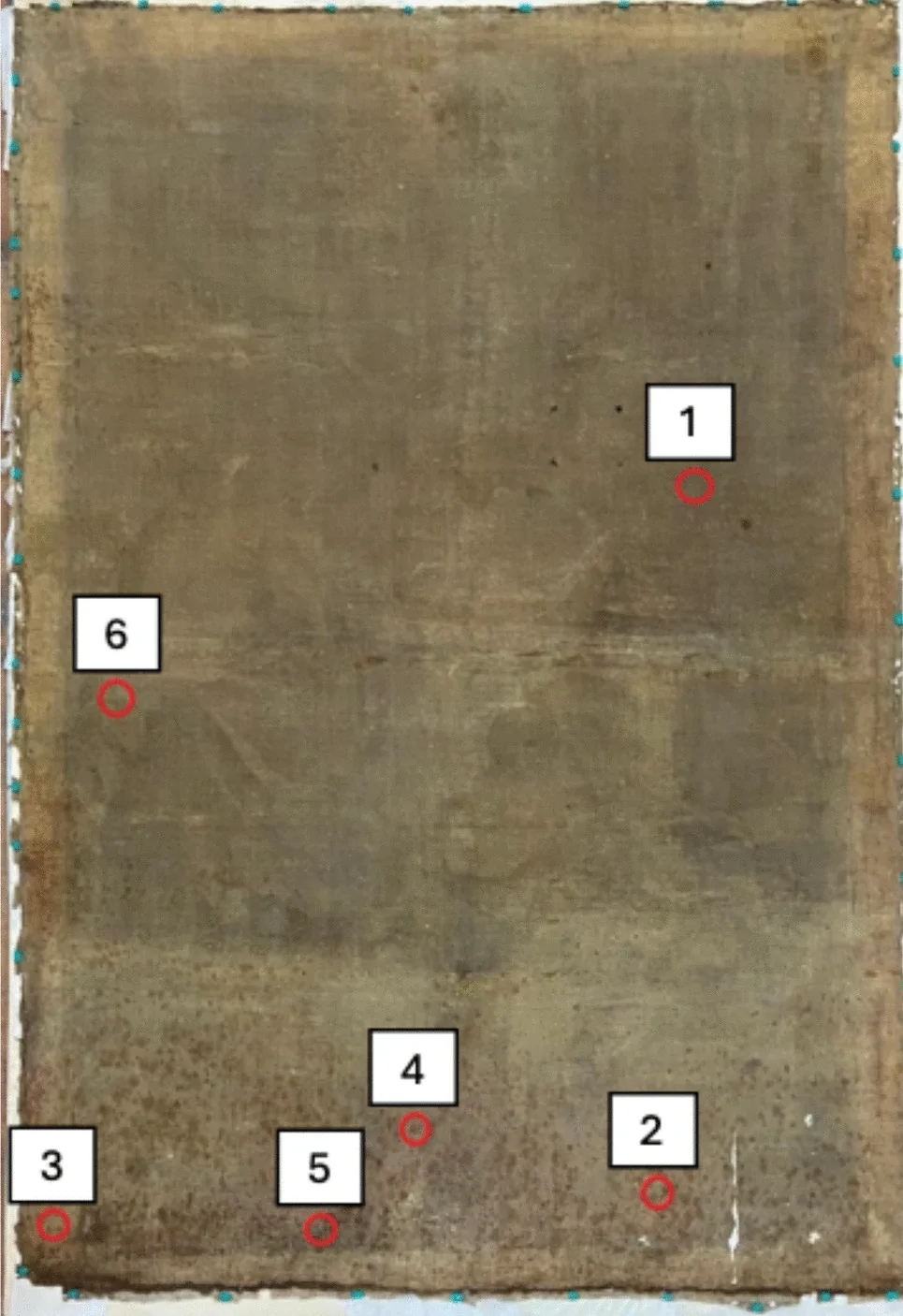
Sampling points on the back of the canvas

Sampling was performed on the back of the original canvas in six distinct points (Fig. 2). Point 1 coincided with an apparently not altered area, points from 2 to 5 corresponded to lower area of the canvas that showed a greater deterioration, and finally, point 6 was located below the canvas support structure. The biological material was collected through contact plate method, using tryptic soy agar (TSA) (g L^−1^: casein peptone 17.0, soya peptone 3.0, NaCl 5.0, K_2_HPO_4_ 2.5, glucose 2.5, agar 15.0) and Sabouraud dextrose chloramphenicol agar (SAB) (g L^−1^ of medium casein peptone 5.0, meat peptone 5.0, glucose 40.0, chloramphenicol 0.5, agar 15.0). The TSA medium was used for both bacteria and fungi isolation, while the SAB medium was specifical for fungi. Afterwards, the culture plates were incubated at 28 °C for 5 days and different colonies, based on their morphological differences (i.e., shape, size, color, etc.), were picked up and streaked on the same media until they were purified to homogeneity, isolating, in this way, 17 bacterial and six fungal strains, respectively. The isolated microorganisms were then characterized morphologically.

### DNA extraction, amplification, and sequencing of 16S rDNA and 18S rDNA

The DNA extraction of isolated bacteria and fungi was performed by means of REDExtract-N-Amp™ Tissue PCR kit (Merck Life Science Srl, Milan, Italy) following the supplier instructions.

The region V4-V5 of 16S rDNA gene was amplified with bacterial universal primers com1 (5′ CAGCAGCCGCGGTAATAC 3′) and com2 (5′ CCGTCAATTCCTTTGAGTTT 3′). While the region ITS2 of fungal 18S rDNA gene was amplified with primers ITS3 (5′ GCATCGATGAAGAACGCAGC 3′) and ITS4 (5′ TCCTCCGCTTATTGATATGC 3′). PCR volume (20.0 μL) contained 2.0 μL of DNA solution, 2.0 μL of 10X Red Extract-N-Amp_PCR_ Ready Mix of the Sigma kit (Sigma, Milano-Italy), 1.0 uM of each forward and reverse primer. PCR conditions were as follows: initial denaturation for 3 min at 94 °C, 35 cycles of denaturation at 94 °C for 60 s, annealing at 55 °C for 60 s, and elongation 120 s at 72 °C and, at end of the cycles, an additional final elongation step of 7 min at 72 °C. Finally, the amplicons sequencing was performed by Eurofins Genomics (Ebersberg, Germany), and they were identified by a similarity search using the BLAST function of GenBank at the National Center for Biotechnology Information (NCBI) electronic site (NCBI. Available online: http://www.ncbi.nlm.nih.gov/, 07 March 2025). Moreover, the sequences were deposited in GenBank (05 April 2025) under accession numbers reported in Table 1.

**Table 1:** Microbial identification

Isolates	Acc. no. of isolates	Closest relative taxa	Acc. no. of the closest relatives	Query cover	Sequence identity
1B2	*	*Kocuria rhizophila*	LN714837.1	98%	100%
1Or	PV444376	*Arthrobacter agilis*	KC788050.1	97%	99.73%
2A	PV444391	*Staphylococcus equorum*	OM919522.1	97%	99.46%
2B	PV444398	*Pseudomonas indoloxydans*	MH910788.1	99%	97.59%
2C	*	*Micrococcus luteus*	OR975986.1	100%	100%
45B	PV444401	*Pseudomonas stutzeri*	HF571100.1	98%	99.47%
4C	PV444438	*Staphylococcus saprophyticus*	KJ634143.1	100%	99.72%
4Ea	*	*Bacillus licheniformis*	OL873154.1	100%	100%
4H	*	*Arthrobacter* strain NR13	MG835286.1	99%	100%
5A	PV444402	*Kocuria carniphila*	AM237385.1	100%	99.59%
5B	*	*Bacillus pumilus*	EF467045.1	97%	100%
5D	PV444405	*Micrococcus luteus*	KC581937.1	98%	99.47%
5Ea	*	*Staphylococcus aureus*	GQ214333.1	100%	100%
5Eb	*	*B. licheniformis*	JX237842.1	100%	100%
6Ba	*	*Priestia endophytica*	JQ831507.1	98%	100%
6C	PV444437	*Bacillus subtilis*	KC549671.1	99%	99.73%
6E	PV444410	*Kocuria rosea*	MW033920.1	99%	99.73%
F1G	PV444434	*Cladosporium cladosporioides*	OM237311.1	100%	99.31%
F2A	PV444436	*Penicillum chrysogenum*	MW826163.1	99%	99.73%
F3A	*	*Aspergillus niger*	OK091639.1	99%	100%
F3B	PV444442	*P. chrysogenum*	MW826163.1	100%	99.67%
F4B	PV444441	*P. chrysogenum*	MK254989.1	99%	99.37%
F6A	PV444457	*P. chrysogenum*	JF922035.1	100%	99.36%

“*” Accession number was not inserted in database for sequences with 100% identity

The isolated strains reported in Table 1 are preserved in our collection (stored at − 80 °C at the Department of Chemistry and Biology of the University of Salerno) and will be available for everyone who wants them, contacting the Dr. Gianmaria Oliva.

### Measurement of painting pigments deterioration

Two pigments were chosen for biodeterioration tests: yellow ochre 0324 (FeO(OH), CaCO_3_, CaSO_4_, CTS Conservation, Italy) and ivory black 0597 (animal bone ash and PO_4_^2−^, CTS Conservation, Italy). The bacterial growth, in the presence of each pigment and their capability to induce pigment deterioration, was tested on Bushnell-Hass (BH) medium (g per 1 L of medium: MgSO_4_⋅7H_2_O 0.2, NH_4_NO_3_ 1.0, CaCl_2_ 0.02, glucose 1.0, K_2_HPO_4_ 1.5, KH_2_PO_4_ 1.0, pigment 1.0). Contrarily, fungal activity was evaluated on modified metal toxicity (mMT) medium (g L^−1^ of medium: MgSO_4_⋅7H_2_O 1.0, NH_4_Cl 1.0, CaCl_2_ 0.06, Na_2_SO_4_ 2.13, glucose 10.0, yeast extract 0.05, tryptone 0.5, pigment 1.0). A cell suspension of 1.0 optical density at 600 nm (OD_600_), for each microbial sample, was prepared in saline solution (NaCl 0.9% w/v) from fresh microbial culture. Afterwards, 3 mL of liquid medium (BH or mMT) were added in 15-ml Falcon tubes, and 30 μL of each cell suspension were inoculated into respective tubes (final OD_600_ = 0.01). Each sample was prepared in triplicate and was incubated for 5 days at 28 °C in orbital shaker at 200 RPM (New Brunswick Innova 43, Edison, NJ; USA).

In bacterial culture, it was evaluated; the pigment color change. In particular, the tubes were centrifugated at 6000 RPM for 10 min; the pH of supernatant was estimated with a pHmeter (S-610L, Peak Instrument, China) and then it was discarded; therefore, the precipitate was recovered and diffused homogeneously on a slide, and it was air dried. At the end, color changes were assessed with a colorimeter (Datacolor Italia s.r.l, Monza, Italy) measuring the chromaticity coordinates L*, a*, and b* and estimating the Δ*E* value (absolute chromatic variation) (Mao et al. 2024) defined as follows:

$$\Delta E= \sqrt{{(\Delta L)}^{2 }+ {(\Delta a)}^{2 }{+(\Delta b)}^{2}}$$

Instead, the fungi incorporated the pigment into the mycelia; therefore, it was not possible to evaluate the colorimetric alteration as in the case of bacteria. About that, it was estimated the pigment amount incorporated in fungal biomass, measuring the difference between the initial pigment amount and that still present in solution after the fungal growth. Therefore, 1 mL of culture solution was transferred in a new tube (2.0 mL) avoiding the fungal aggregates. The tubes were centrifugated at 12,000 rpm for 5 min, the supernatant was discarded, and the samples were oven dried at 70 °C for 48 h. Finally, the dry weight of pigment was determined.

### Preparation of plant extracts

Initially, three plant species known for their proven antimicrobial properties were selected: rosemary (*Salvia rosmarinus* Spen 1835); garlic (*Allium sativum* L.); and lemon thyme [*Thymus citriodorus* (Pers.) Schreb.].

The same hydroalcoholic extracts were prepared for either rosemary or lemon thyme: 10 g of fresh leaves were homogenized in 20 mL of solution (1:1, water:ethanol 100%) in a mortar at room temperature. Instead, the garlic extract was prepared by homogenizing 10 g of fresh bulbs in 20 mL of water in a mortar. All extracts were filtered through a 0.45-μm porous membrane and then stored at 4 °C in the dark.

### Evaluation of antimicrobial activity

The antimicrobial activity of the three natural extracts was evaluated on all isolated bacteria and fungi from the canvas. Hence, 50 μL of fresh cell suspension (0.5 OD_600_) of each bacterial culture, suspended in saline solution (0.9% NaCl), were diffused on Luria–Bertani (LB) agar medium. Then, 3.0 μL of each natural extract were spotted on the agar plate. A drop of extraction solvent was used as control. Finally, the plates were incubated at 28 °C for 48 h. The formation of an inhibition halo proved the effective antimicrobial of the assayed extract.

### Evaluation of antimicrobial activity on canvas and different methods of application

The antimicrobial activity of rosemary extract was evaluated on five strains previously isolated from the original canvas and inoculated on virgin linen canvas (canvas mock-up dimension: 12 × 12 cm) (Supplemental Fig. S2A), as preliminary evaluation of the extract’s ability to inhibit the growth of selected microorganisms when applied to a textile support. In this regard, four strains sensitive to rosemary extract (1OR, 4Ea, 5Eb, 6E) and one resistant strain (4C) were selected.

A canvas sample was placed in Petri dishes (∅140 mm) and superficially sterilized with UV radiation for 15 min. Then, for each microorganism, 5.0 μL of fresh cell suspension were spotted on canvas. Suspensions were prepared from overnight grown cultures by diffusing the bacteria in saline solution (0.9% NaCl) at density of 0.5 OD_600_. After 1 h, microbial contamination was evaluated by means of contact plate method, using TSA as a growth medium, then plates were incubated at 28 °C for 5 and 10 days. Afterwards, the canvas was treated by means of 2.0-mL rosemary extract spraying it directly on the canvas surface (0.03 mL cm^−2^), repeating the application three times, 1 h apart. The canvas was air dried, and microbial survival was assessed using both TSA and SAB agar plate, as described above.

Further analyses were conducted on the old lining canvas of the painting (canvas mock-up dimension: 12 × 12 cm), removed during the conservation operations (Supplemental Fig. S2B), and the efficacy of rosemary extract against the microorganisms already present on it was evaluated. The lining canvas sample was chosen due to its close resemblance to the structure and characteristics of the original ancient canvas.

Also in this case, microbial contamination was evaluated pre- and post-treatment with rosemary extract, with contact-plate method, as indicated above.

Other two strategies of antimicrobial application were also evaluated. The first was the extract application through Evolon® tissue (Cts Conservation, Vicenza, Italy). Evolon*®* is an innovative technical tissue obtained from a mixture of polyester and polyamide microfilaments, which give at the material unique properties such as resistance, breathability, and lightness (Vergeer et al. 2019). Thanks to its structure without warp and weft, it is particularly suitable for several applications including material disinfection. Furthermore, it is a sustainable material, as it is recyclable and free of chemical solvents. The Evolon*®* tissue was immersed for 2 min in the hydro alcoholic rosemary solution, then wrung out and placed for 1 h on the canvas under a weight of about 2 kg so to have an its uniform distribution on the canvas. As previously described, biological material (microorganisms) was sampled before and after treatment in the same way as above described.

The second one was the nebulization on the pretreated canvas with cyclomethicone D5. This compound is a hydrophobic solvent used in conservation to reduce temporally the sensitivity of porous material to water. A control sampling was performed before treatment. Subsequently, 2 mL cyclomethicone D5 was sprayed on the canvas (0.03 mL cm^−2^) and air dried for 2 h. After that, rosemary extract was nebulized on canvas, and the application was repeated three times, 1 h apart as above. The treated canvas was air dried, and then the microbial community was collected through contact plate method, using TSA agarized media.

Finally, the possible impact of rosemary extract, on the optical properties of the cellulose support, was also evaluated through colorimetric analysis before and after the treatment. For this purpose, the extract was sprayed on a piece of virgin canvas (as above), and it was measured the Δ*E* value (absolute chromatic variation).

## Results

### Analysis of textile fibers

In the case of original canvas, microscopical analysis highlighted that in both samples TO1 and TO2, the individual fibers showed a thin and uniform structure (Supplemental Fig. S3A-B). They had a cylindrical shape with a thin central duct and a sharp end. Furthermore, they had clear transverse striations that crossed each other obliquely forming an “x” shaped configuration, while showing a slight dilation at almost regular intervals. Based on the observed characteristics, we deduced that these fibers belonged to the linen species. In the case of relining canvas, it was observed that TR1 sample showed the same characteristics previously described in the original canvas (Supplemental Fig. S3C). For the sample TR2, the observation revealed important details (Supplemental Fig. S3D). In fact, some fibers were like linen, while other appeared more robust, cylindrical, and slightly flattened, with longitudinal and irregular transverse striations. These streaks crossed each other frequently to form an “x” shaped configuration but were free of swelling. Based on these observations, it was possible to hypothesize that the sample examined was composed from a mixture of linen and hemp fibers. In conclusion, the analysis of textile fibers highlighted the natural origin of both canvases.

### Preliminary screening with ultraviolet fluorescence photography

**Fig. 3:**
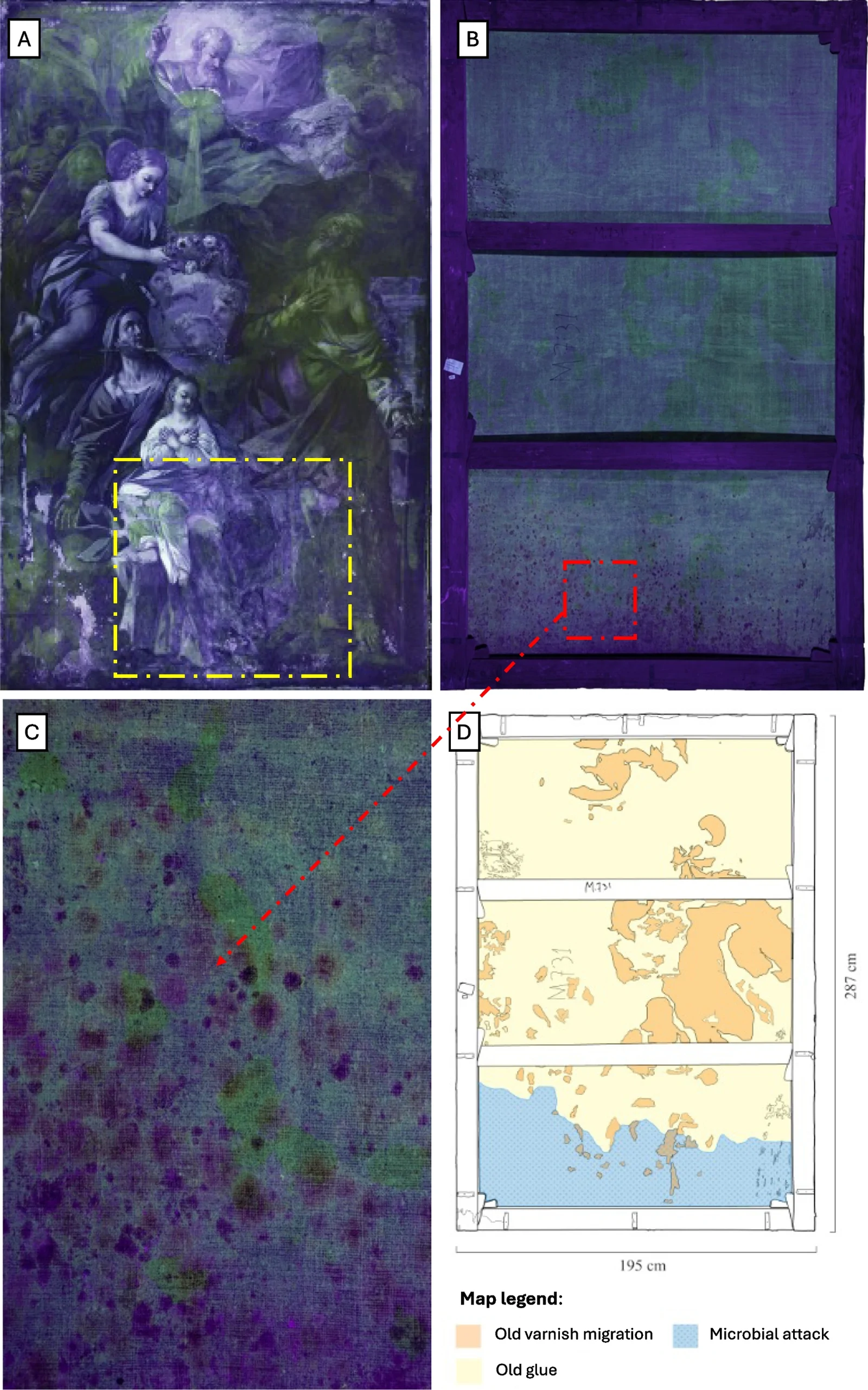
Ultraviolet fluorescence photography. **A** Front side of the canvas exposed to ultraviolet fluorescence; yellow rectangle indicated whitish fluorescence in the lower part of the painting. **B** Back side of the canvas exposed to ultraviolet fluorescence. Red rectangle highlighted a part of canvas where we hypothesized a microbial contamination. **C** Possible microbial contamination highlighted by orange-brown dots. **D** Deterioration map estimated by ultraviolet fluorescence photography analysis

The UV fluorescence analysis conducted revealed a whitish fluorescence in the lower part of the painting (Fig. 3A–C). Moreover, the acquired images of the back showed a fluorescence ranging from orange to brown in correspondence of possible microbiological contamination (Fig. 3B, C). Areas with a yellow-green fluorescence were present on the back, due to the amber varnish that migrated during previous restorations, and the old relining paste was also observed (Fig. 3B–D). The glue used commonly produces a yellow-whitish fluorescence (Fig. 3B–D), that is not too much intense, probably an indication that the glue was not present in large quantities between the original canvas and the one to be relining.

### Microbial contamination and community characterization

**Fig. 4:**
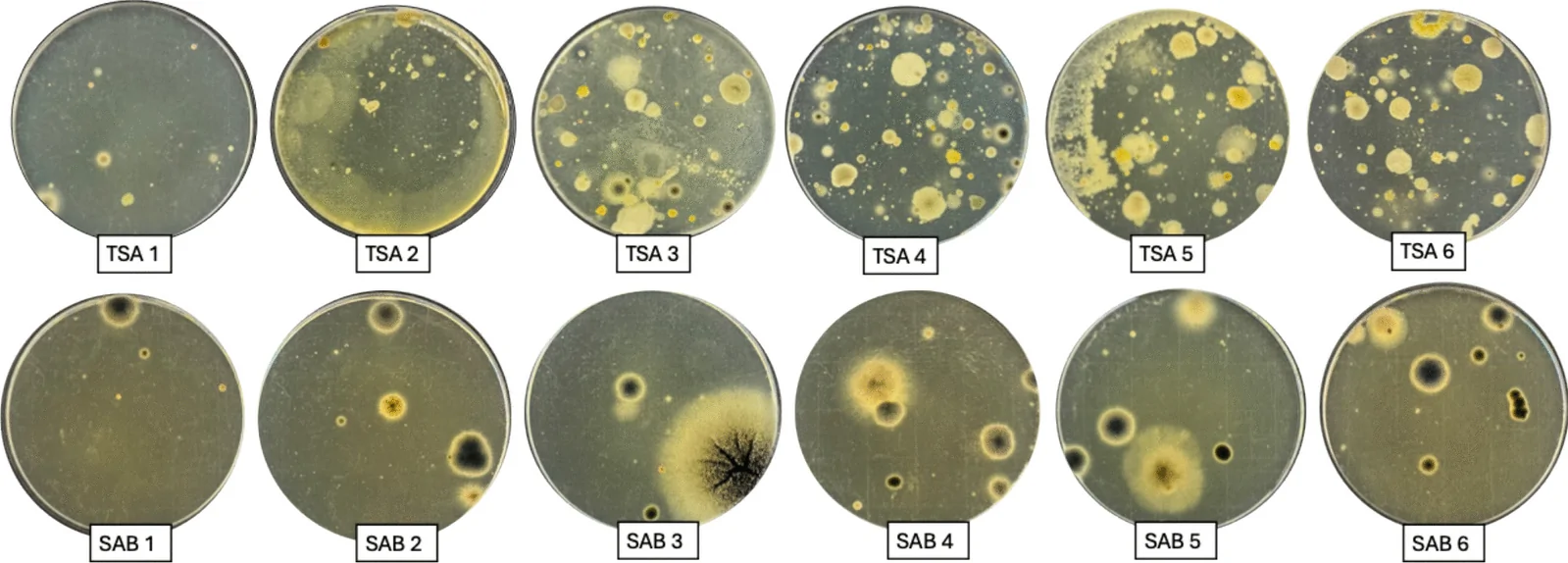
Contamination grade in different sampling points of the canvas. Samplings with TSA represent both fungal and bacterial contamination, while those with SAB only represent fungal contamination

The contact plate method highlighted a significant microbiological contamination of either bacterial or fungal in all sampling points, except for those where the canvas did not apparently show any alterations (Fig. 4).

The morphologically different colonies were collected and purified to homogeneity until 17 bacterial and six fungal strains were obtained. In the case of bacteria, 15 strains were Gram-positive and two were Gram-negative (Supplemental Fig. S4). The alignment of the 16S rDNA sequences recognized seven different bacterial genera: *Kocuria*, *Micrococcus*, *Arthrobacter*, *Staphylococcus*, *Bacillus*, *Priestia*, and *Pseudomonas* (Table 1). The most represented genus was *Bacillus* (phylum *Firmicutes*, family *Bacillaceae*), with four strains, phylogenetically closer to: *Bacillus licheniformis*,* Bacillus pumilus*, and* Bacillus subtili*s (sequence identity between 99.73 and 100%). It was followed by *Kocuria* (phylum *Actinomycetota*, family *Micrococcaceae*) and *Staphylococcus* (phylum *Firmicutes*, family *Staphylococcaceae*), each with three strains, closer to distinct species: *Kocuria rhizophila*, *Kocuria carniphila*,* Kocuria rosea* (sequence identity 99.6–100%) and *Staphylococcus equorum*, *Staphylococcus saprophyticus*, *Staphylococcus aureus* (99.5–100%), respectively. The six fungal strains were identified both microscopically and molecularly. The ITS DNA sequences were compared with those available at NCBI Data Base and were recognized to belong to three different species: *Aspergillus niger*, *Penicillum chrysogenum*, and *Cladosporium cladosporioides* (99.3–100% identity).

### Interaction of microorganisms with pigments

**Fig. 5:**
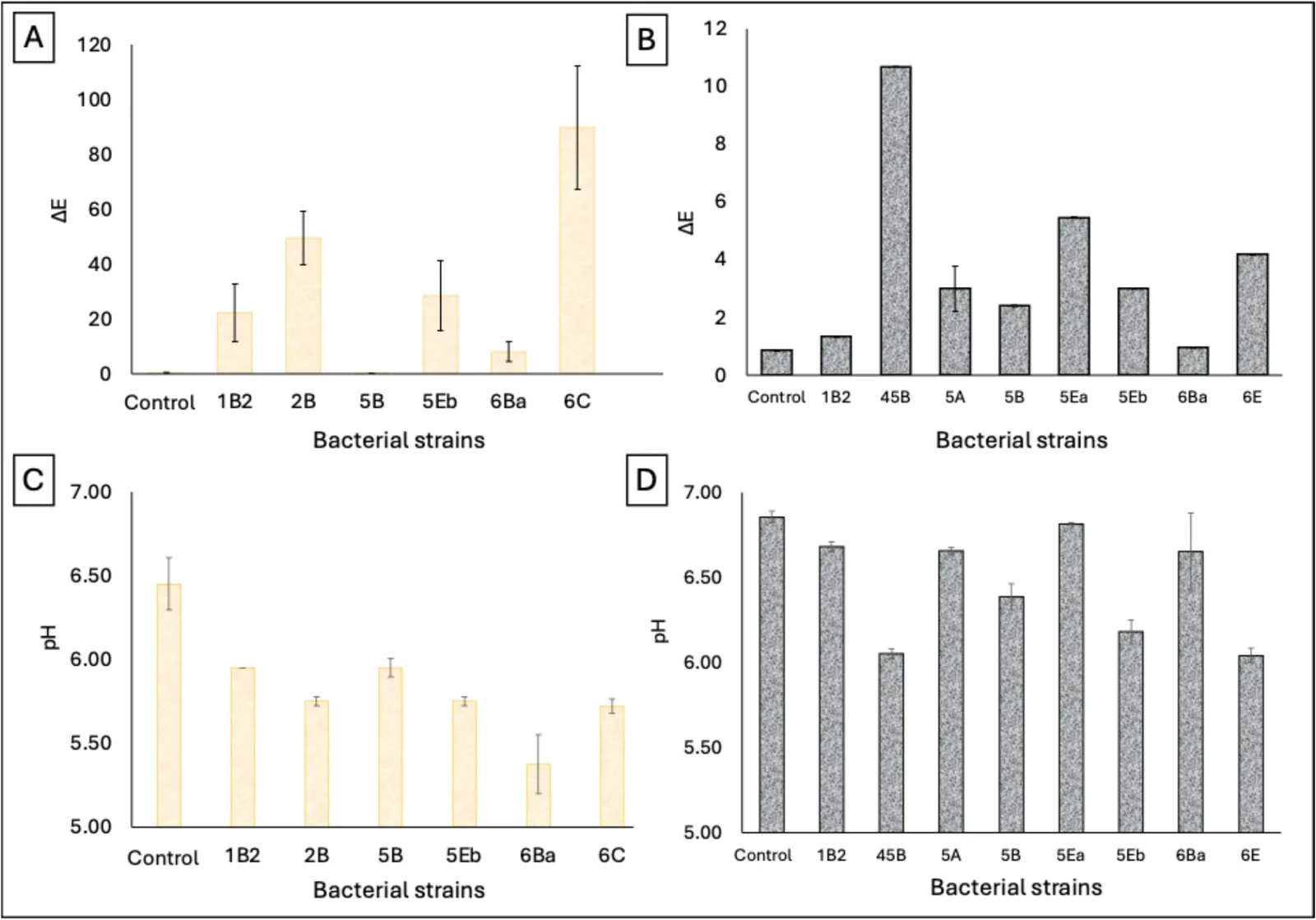
**A**–**B** Evaluation of color changes estimating the Δ*E* value (absolute chromatic variation) in the case of yellow ochre (**A**), or ivory black (**B**). **C**–**D** pH values of the culture medium following the microbial growth, in the case of yellow ochre (**C**), or ivory black (**D**)

The study of the interaction between bacteria and pigments was conducted only on a part of the isolated strains (1B2, 2B, 5B, 5Eb, 6Ba, and 6 C for yellow ochre; 1B2, 45B, 5 A, 5B, 5EA, 5 EB, 6Ba, and 6E for ivory black), chosen based on their ability to grow in the experimental conditions (BH culture medium in the presence of selected pigments). On this basis, a total of 11 bacterial strains were employed for further investigations. In the presence of yellow ochre, six bacterial strains were grown; and four of them, 1B2, 2B, 5Eb, and 6Ba showed the capability of physically interact with pigment through the formation of aggregates (Supplemental Fig. S5). Moreover, the determination of the absolute chromatic variation (Δ*E*) evidenced for 6 C strain the highest value (89.78 ± 22.47), indicating a severe chromatic alteration (Fig. 5A). A less severe but still evident color variation was caused by 1B2, 2B, and 5Eb (49.51 ± 9.89, 28.51 ± 12.67, and 22.29 ± 10.41, respectively), while 5B and 6Ba showed Δ*E* values lower than 10.

In the case of ivory black, eight bacterial strains were grown, and six of them physically alter the pigment (Supplemental Fig. S6). However, the chromatic variations were less evident (Fig. 5B). The highest value of Δ*E* was observed for the 45B strain (10.67 ± 0.01), indicating a slightly alteration, while for the others were observed values less than 6.0.

At the same time, the pH of culture medium of either yellow or black pigment was evaluated. In the case of yellow ochre, the pH decreased for all strains (< 6.00 for all vs 6.45 ± 0.16 of control sample without bacteria). The lowest pH value was observed in the strain 6Ba (5.38 ± 0.18) (Fig. 5C). In the case of ivory black, the pH values of strains 1B2, 5 A, 5Ea, and 6Ba (all around 6.60) were slightly lower than control (6.86 ± 0.04), while the strains 45B, 5B, 5Eb, and 6E showed more acidic values (between 6.00 and 6.30) (Fig. 8D).

**Fig. 6:**
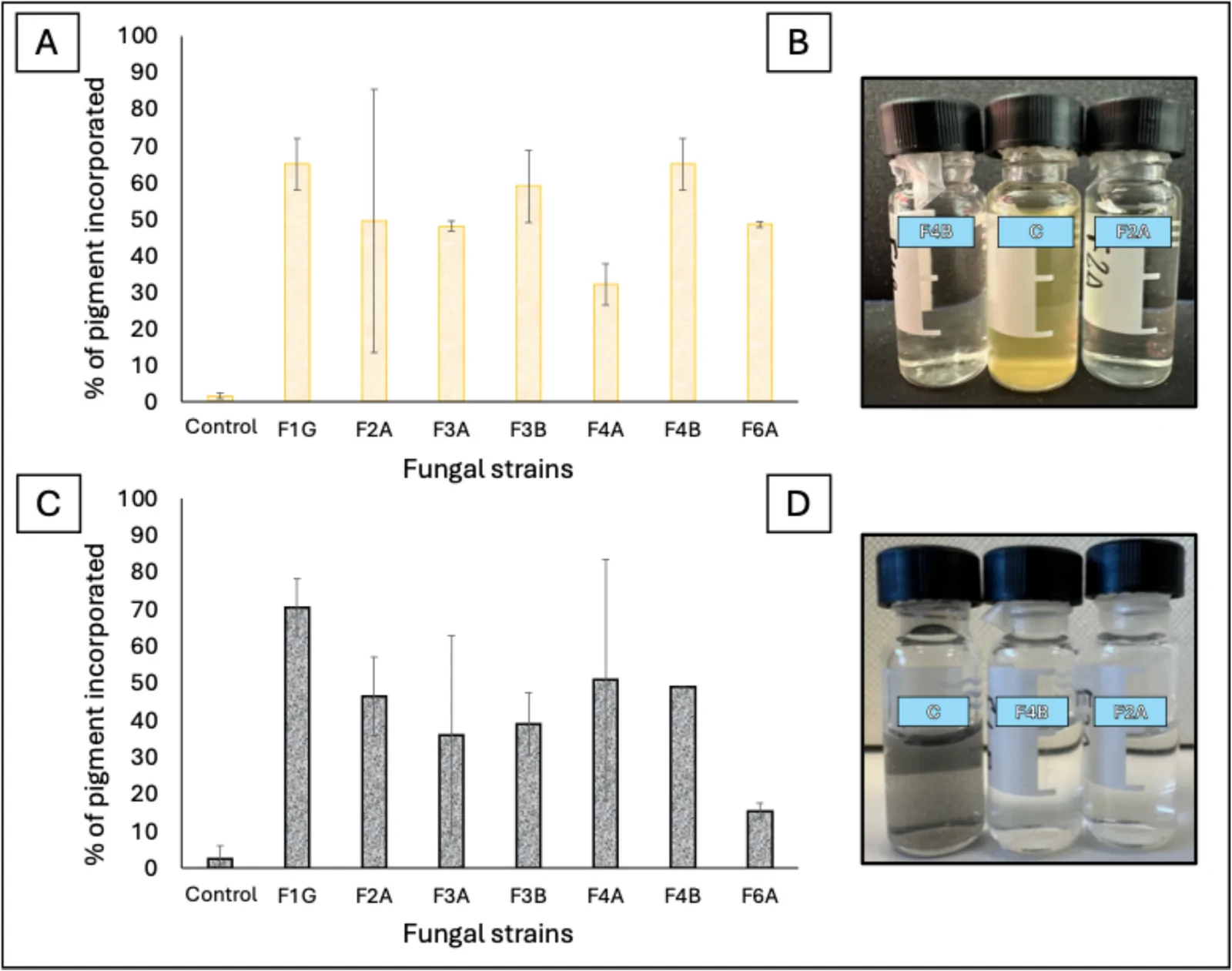
Percentage of pigment yellow ochre (**A**) and ivory black (**C**) trapped/incorporated in the fungal biomass. Culture media collected avoiding the fungal biomass, of the Control, F4B, and F2A strains grown in the presence of yellow ochre (**B**) or ivory black (**D**)

On the other hand, all fungal isolated were able to grow in the presence of both pigments. However, for all strains, a considerable capacity for pigment sequestration in the mycelium was observed (Supplemental Fig. S7). For this reason, it was not possible to evaluate the colorimetric alteration as for bacteria, and we restricted the analysis to the determination of the accumulation capacity in the biomass at the end of their growth in the culture medium. In the presence of either yellow ochre or ivory black, the F1G and F4B strains showed the greatest capability to incorporate the pigments (~ 70% of pigment was incorporated) (Fig. 6A–C), and Fig. 6B–D shows the presence of the yellow ochre and ivory black pigment in the culture medium (devoid of mycelium) recovered at the end of growth.

**Fig. 7:**
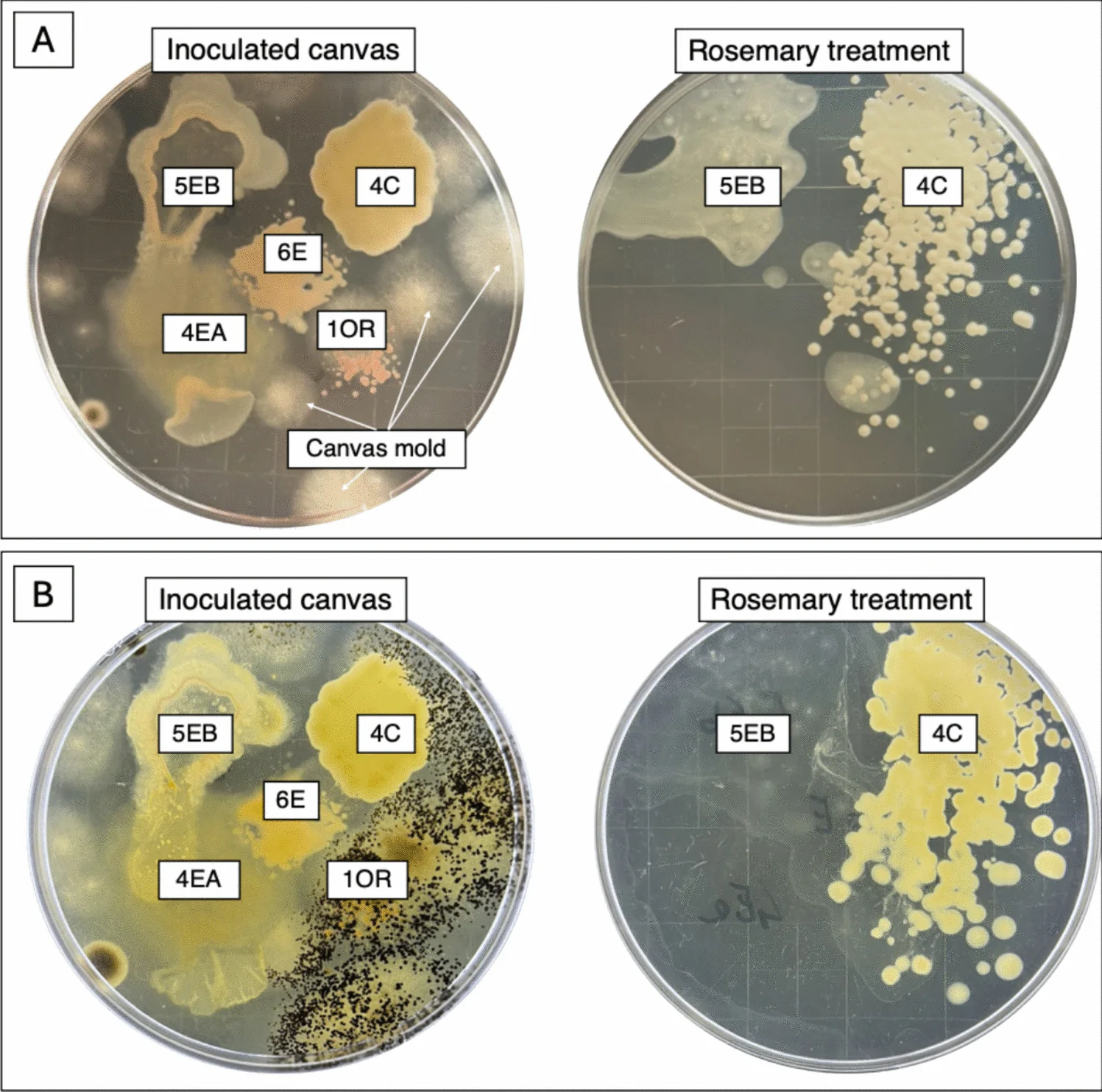
Comparison between the samples obtained from the virgin linen canvas, inoculated with five bacterial strains, before and after rosemary treatment, at 5 (**A**) or 10 growth days (**B**) in TSA medium. At 10 days of the growth, some molds were detected in the untreated. The presence of molds is probably due at a secondary contamination of UV-treated canvas or a low efficacy of the treatment against microorganisms present in the weaving of the sample

Figure 7A-B shows the results on the virgin linen canvas. Treatment with rosemary extract inhibited effectively the growth of sensitive strains 1OR, 4Ea, and 6E, but not 5Eb, probably because the duration of treatment (1 h) was insufficient for complete inhibition of this strain, while it was ineffective against the resistant strain 4C

### Antimicrobial activity evaluation of natural extracts

The antimicrobial activity of three natural extracts was assessed on all the isolated bacteria and fungi, evaluating the inhibition of growth on agar medium, through the formation of the inhibition halo. The rosemary extract showed the higher antimicrobial activity, inhibiting the growth of 13 bacterial strains (76% of tested population) (Table 2, Supplemental Fig. S8), while garlic extract showed intermediate effects, inhibiting the growth of six bacterial strains (35%). Finally, thyme extract was the least active, inhibiting only one bacterial strain (6%). Moreover, it was observed that only rosemary extract inhibited the growth of *P. chrysogenum* strains (F2A, F3B, F4B, F6A).

**Table 2:** Antimicrobial activity of rosemary, garlic, and thymus extracts against the isolated bacteria and fungi; “ + ” growth inhibition, “ − ” no antimicrobial activity

Strain	Rosemary	Garlic	Thymus
1B2	** + **	** + **	** + **
1Or	** + **	** + **	** − **
2A	** − **	** − **	** − **
2B	** + **	** + **	** − **
2C	** + **	** − **	** − **
45B	** + **	** + **	** − **
4C	** − **	** − **	** − **
4Ea	** + **	** − **	** − **
4H	** − **	** + **	** − **
5A	** + **	** − **	** − **
5B	** + **	** − **	** − **
5D	** + **	** − **	** − **
5Ea	** − **	** − **	** − **
5Eb	** + **	** − **	** − **
6Ba	** + **	** − **	** − **
6C	** + **	** − **	** − **
6E	** + **	** − **	** − **
F1G	**-**	** − **	** − **
F2A	** + **	** − **	** − **
F3A	** − **	** − **	** − **
F3B	** + **	** − **	** − **
F4B	** + **	** − **	** − **
F6A	** + **	** − **	** − **

### Antimicrobial activity evaluation on canvas

Based on the preliminary analysis, rosemary extract was the most effective to inhibit the growth of the canvas-isolated microorganisms; therefore, it was chosen for the application on canvas samples. First, the antimicrobial efficacy of the extract was assessed against five of the bacterial strains isolated from the original canvas (four sensitive and one resistant), spotted on pieces of virgin linen canvas superficially pre-sterilized with UV. The application was done by spraying the extract on the surface of the sample. Then, the extract was also evaluated against the microbial species already present on the relining canvas of the painting (bacteria and fungi). In the latter case, three different application methods, optimized for the conservation of this kind of artworks, were tested. The first treatment included the direct spraying on the canvas surface. The other two were calibrated to limit the penetration of the aqueous fraction of the extract in the substrate, pre-treating the canvas surface with the hydrophobic compound cyclomethicone D5, or by applying the extract through Evolon*®* tissue (see Materials and methods section).

**Fig. 8:**
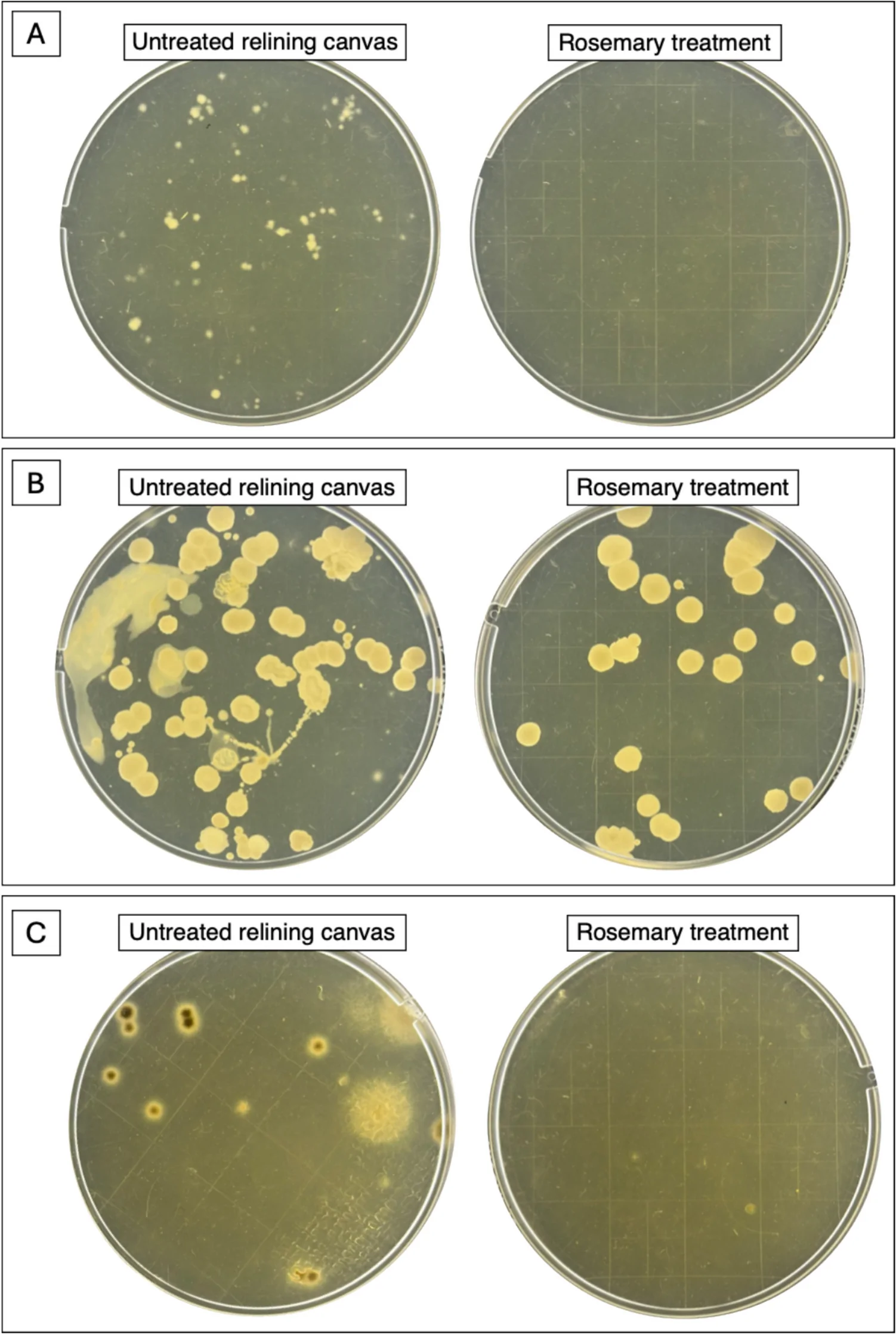
Comparison between the bacterial contamination sampled from the old relining canvas, before and after rosemary treatment, at 24 h (**A**) and at 48 h (**B**) of growth in TSA medium; and comparison between fungal contamination, before and after rosemary treatment, at 7 days of growth in SAB medium (**C**)

**Fig. 9:**
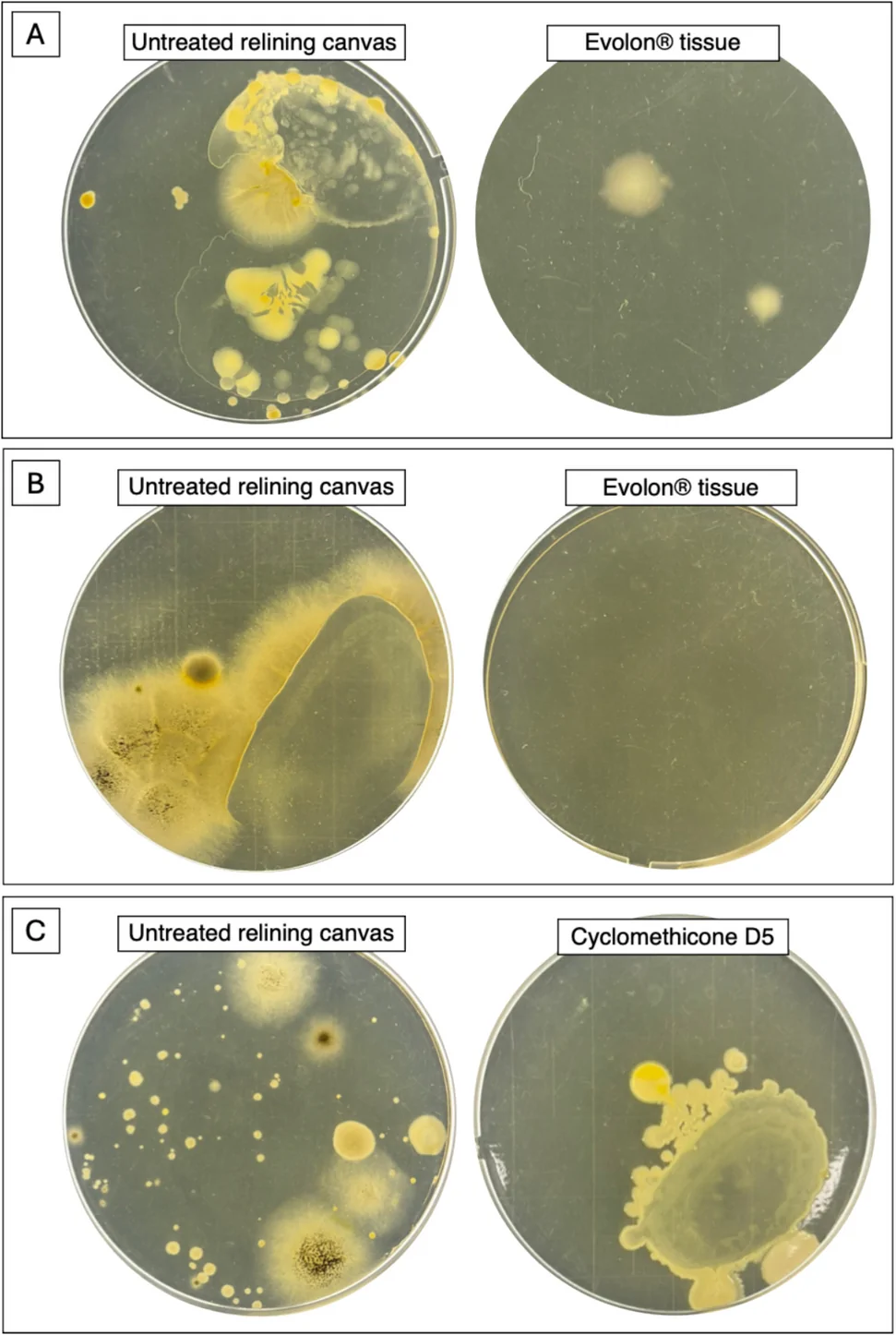
**A** Comparison between the bacterial contamination sampled from the old relining canvas, before and after Evolon® application, at 48 h of growth in TSA medium; and **B** comparison between fungal contamination, before and after the same treatment, at 7 days of growth in SAB medium. **C** Comparison between the microbial contamination before and after the pre-treatment with cyclomethicone D5, after 5 days of growth in TSA medium

On the other hand, by spraying antimicrobial on the relining canvas surface, a 100% reduction of bacterial growth (Fig. 8A) was revealed at 24 h of incubation, and approximately 75% after 48 h (Fig. 8B). The growth of the fungal population resulted completely inhibited until the end (7 days) (Fig. 8C). Similar results were obtained with the application of Evolon*®* tissue (Fig. 9A–B). Finally, in the canvas pre-treated with cyclomethicone D5, it was observed a less efficiency of the extract, probably due to its difficulty to penetrate the hydrophobic surface (Fig. 9C).

A common problem in the use of the natural extracts is the chromatic alteration of the cellulose support due to the treatment. Therefore, for completeness, we also evaluated the suitability of the antimicrobial by measuring the Δ*E* value on canvas before and after its application. The result did not show any significant chromatic variation (Δ*E* = 0.26 ± 0.02), confirming its suitability for the artifact treatment.

## Discussion

Biodeterioration of canvas paintings is a complex process influenced prevalently by environmental conditions, conservation practices, and macro- and micro- organism activities. Factors such as humidity, temperature, and the presence of organic materials used in conservation or as binders, contribute significantly to microbial growth and subsequent biodeterioration of artworks. In this study, we analyzed the microbial role in the deterioration of an eighteenth century painting signed by an Italian artist of the late Baroque: D. Carlo Malinconico. The painting: “Sant’Anna, San Gioacchino e la Vergine bambina,” most likely belonged to the Monastery of San Giovanni Battista delle Monache*,* and was later included in the artistic heritage of the Gallery of Naples Academy. Due to long stay in the warehouse (more than 70 years) where the climatic conditions were changed continuously with high variations of humidity and temperature, the painting was in a poor state of conservation with structural damages as support deformation, loss of adhesion of the paint film, and a microbial attack visible on the back of the lower part of the canvas, where many purple-red-white spots were present. Corresponding spots are observed also on the relining canvas, highlighting a similar microbiological contamination for both the canvases.

The whitish fluorescence highlighted in UV fluorescence analysis could be attributable to the damage caused by humidity, or to the effects of a microbiological contamination present on the back (Triolo 2020). Moreover, the orange-brown fluorescence observed can also be produced by the metabolism of both fungi and bacteria as exudates able to degrade the painting (Pavić et al. 2015). This deterioration could be due to the combined action of the high humidity and the organic materials. In particular, the microbiological attack was localized at the lower part of the artwork where the painting suffered damage from rising damp, a phenomenon for which water rises from the ground along the building walls where it is preserved. About that, Rubio and Bolívar (1997) observed the synergistic effects of fungi and bacteria in creating chromatic and structural alterations, particularly under high humidity conditions. The purple-red-white spots observed on the artwork could be due to pigment-producing bacteria. Compound as astaxanthin, canthaxanthin, prodigiosin appear typically of red color and are produced by several microorganisms as *Agrobacterium aurantiacum*, *Alteromonas rubra*, *Serratia marcescens* (Lee 2025). Then, violacein and zeaxanthin molecules appear of purple and brown color and are produced by bacteria of genera *Bacillus*, *Brevibacterium*, or *Staphylococcus* (Abdulkadir 2017). Fungi also are involved in pigments production and chromatic alteration of artworks. *C. cladosporioides* and *P. chrysogenum*, both isolated in the present study, are known to produce melanins and other dark pigments that can penetrate paint layers, causing persistent color alterations (Sterflinger and Piñar 2013). In this regard, fungal melanins are blackish-brown pigments that accumulate in cell walls and can diffuse into porous materials such as canvas, causing brown or purple spots.

Santos et al. (2009) investigated the microbial community structure of old paintings, demonstrating that nutrient-rich environments are an ideal environment for development of different detrimental microbial communities, able to lead at a visible degradation. Therefore, our study focused on microbial communities colonizing canvas, their interaction with the analog most representative pigments, and evaluation of the applicative potential of natural antimicrobial agents for conservative treatment of the paintings.

The genetic characterization of microbial strains isolated from the canvas, revealed the presence of both bacterial and fungal strains. Seventeen bacterial isolates were identified by means of 16S rDNA analysis, phylogenetically belonging to 14 species clustered into seven genera: *Kocuria*, *Micrococcus*, *Arthrobacter*, *Staphylococcus*, *Bacillus*, *Priestia*, and *Pseudomonas*. Many of these species have already been associated with the biodeterioration of cultural heritage. In the most represented genus *Bacillus*, *B. licheniformis* and *B. subtilis* are known for producing enzymes and organic acids that degrade both binders and pigments (Phulpoto et al. 2016; Pandey and Kiran 2020). These species also exhibit metabolic versatility, as shown in the studies of Gorbushina (2007), enabling them to colonize harsh environments and accelerate biodeterioration.

*Pseudomonas indoloxydans* and *Pseudomonas stutzeri* are known for their oxidative and reductive enzymatic activities, which affect metal-based pigments, leading to chromatic changes (Pavić et al. 2015). *K. rhizophila* and *M. luteus* were identified as resilient taxa capable of surviving in nutrient-poor conditions (Greenblatt et al. 2004). *Staphylococcus* genus is known for its role in biodeterioration, since some species can secrete lipases and proteases able of degrading organic binders (Parulekar-Berde et al. 2020). In particular, *S. aureus* produces pigments like staphyloxanthin, which may cause chromatic alteration of artworks over time (Askoura et al. 2022).

The isolated fungal species include *A. niger*, *P. chrysogenum*, and *C. cladosporioides*. These microorganisms are recognized for producing extracellular enzymes, such as cellulases and ligninases, able to degrade organic substrates and also excreting some acidic metabolites that solubilize inorganic components (Corbu et al. 2023). About that, *C. cladosporioides*, frequently isolated from biodeteriorated artworks, has been involved in pigment discoloration and loss of pictorial cohesion (Sterflinger and Piñar 2013). *A. niger* is a producer of oxalic acid, which reacts with calcium-based pigments to form insoluble complexes, causing structural damages (Szczepanowska 2023). Thus, the synergistic action of bacteria and fungi causes damages that add up and further deteriorate the artworks sometimes in an irreversible manner. Fungal hyphae physically dislodge pigment particles, while enzymatic activities of both bacteria and fungi alter the chemical composition of binders and pigments.

We demonstrated with our research that the deteriorating ability of the isolated microorganisms alters pigments of the analyzed painting. For yellow ochre, bacterial strains such as *B. subtilis* (6C) and *P. indoloxydans* (2B) caused severe chromatic alterations; in fact, they exhibited the highest ∆*E* values, indicating significant deterioration. Ivory black, while less affected, still showed evident alterations, emphasizing that both organic and inorganic pigments are susceptible to microbial activities. These results confirm the studies of Kujović et al. 2024. The authors report oxidative and reductive changes in metal-based pigments caused by microbial activity. Furthermore, the decrease in pH, observed during microbial growth of our isolates, suggests that acidic metabolites contribute to pigment deterioration, as proved by the marked changes in the case of yellow ochre. About that, other scientific publications clearly demonstrate how organic acid, produced by bacteria, cause pigment destabilization and binder degradation (Ciferri 1999; Di Carlo et al. 2022).

We also demonstrated that the isolated fungal strains incorporate pigments into their mycelial biomass, as in the case of *C. cladosporioides* (F1G) and *P. chrysogenum* (F4B). The pigment-sequestering behavior of our fungal isolates is in accordance with the results of Savković et al. (2021), who defined the physical interaction with pigments, evidenced by the penetration of the hyphal filaments into paint layers, as the primary mechanism of degradation/deterioration. Overall, these findings highlight the capability of fungi to compromise the integrity of the colors of paintings through desegregation and/or the physical sequestration of pigments.

Among the three natural extracts tested, the one of rosemary exhibited the highest antimicrobial activity; in fact, it effectively inhibited 76% of the isolated bacterial strains. This result agrees with prior studies emphasizing the efficacy of rosemary bioactive compounds, including polyphenols and flavonoids, which have powerful antimicrobial properties (Stan et al. 2021; Corbu et al. 2022). Comparable antimicrobial effects of plant-derived biocides on cultural heritage substrates were observed by Russo and Palla (2023). When we applied it on the canvas, the extract showed a severe antimicrobial effect. On virgin linen canvas inoculated with isolated strains, the extract inhibited bacterial and fungal growth effectively, counteracting the bacterial growth contamination. We also explored alternative application methods to optimize rosemary extract use in artifact conservation. Its nebulization onto relining canvas showed a reduction in bacterial growth by ~ 75% within 48 h and 100% of fungal contamination within 7 days. Moreover, the use of Evolon*®* tissue allowed a controlled application of rosemary extract, without reducing its antimicrobial efficiency.

Finally, the canvas pre-treatment with cyclomethicone minimized water-induced swelling or shrinkage, which is particularly relevant for sensitive supports; however, a reduced efficiency of rosemary extracts to counteract microbial contamination was evident. Our approaches to limit biodeterioration of artifacts are in accord with Oriola-Folch et al. (2020), who emphasized the need for customized application techniques to balance efficacy and preservation of original materials. Hence, these results suggest that rosemary extract is a practical, sustainable, and economic alternative to synthetic biocides, providing effective disinfection without any evident damaging of the materials employed by the artist to realize his painting.

## Conclusion

The results of our research demonstrated that certain bacterial and fungal strains isolated from the painting “Sant’Anna, San Gioacchino e la Vergine Bambina” were, in part responsible of its deterioration. These microorganisms interact with pigments resulting in noticeable chromatic alterations of yellow ochre and ivory black confirming that microbial proliferation on the canvas compromises the structural stability of artworks and also significantly affects their visual appearance. Moreover, our work highlights the importance of developing sustainable and minimal invasive approaches for the conservation of cultural heritage. The use of rosemary extract addresses two relevant challenges: environmental sustainability and artwork preservation. Compared to conventional chemical biocides, natural extracts offer an ecological and sustainable alternative, reducing environmental impact and health risks for operators without compromising treatment efficacy. However, it is essential to discuss also their limitations. In fact, our study did not evaluate the long-term efficacy of the treatments under varying environmental conditions, such as fluctuating humidity or temperature. Therefore, we recommend that these treatments be implemented in museum environments with controlled conditions to maximize their effectiveness and longevity. Further studies are required to optimize the formulation and application of these natural extracts, particularly to ensure their effectiveness on a larger scale.

## Supplementary Information

Below is the link to the electronic supplementary material.

ESM 1 (DOCX 26.9 MB)

## Data Availability

No datasets were generated or analysed during the current study.

## References

[CR1] Abdulkadir N (2017) Bacterial pigments and its significance. MOJ Bioequiv Availab 4. 10.15406/mojbb.2017.04.00073

[CR2] Angane M, Swift S, Huang K, Butts CA, Quek SY (2022) Essential oils and their major components: an updated review on antimicrobial activities, mechanism of action and their potential application in the food industry. Foods 11:464. 10.3390/foods11030464 35159614 10.3390/foods11030464 PMC8833992

[CR3] Askoura M, Yousef N, Mansour B, Yehia FAA (2022) Antibiofilm and staphyloxanthin inhibitory potential of terbinafine against *Staphylococcus aureus*: in vitro and in vivo studies. Ann Clin Microbiol Antimicrob 21:21. 10.1186/s12941-022-00513-7 35637481 10.1186/s12941-022-00513-7 PMC9153124

[CR4] Baglioni M, Poggi G, Chelazzi D, Baglioni P (2021) Advanced materials in cultural heritage conservation. Molecules 26:3967. 10.3390/molecules26133967 34209620 10.3390/molecules26133967 PMC8271397

[CR5] Barresi G, Cammarata M, Palla F (2017) Biocide. In: Palla F, Barresi G (eds) Biotechnology and conservation of cultural heritage. Springer International Publishing, Cham, pp 49–65

[CR6] Böhme N, Anders M, Reichelt T, Schuhmann K, Bridarolli A, Chevalier A (2020) New treatments for canvas consolidation and conservation. Herit Sci 8:16. 10.1186/s40494-020-0362-y

[CR7] Camuffo D (2019) Microclimate for cultural heritage: measurement, risk assessment, conservation, restoration, and maintenance of indoor and outdoor monuments. Elsevier, Amsterdam

[CR8] Capodicasa S, Fedi S, Porcelli AM, Zannoni D (2010) The microbial community dwelling on a biodeteriorated 16th century painting. Int Biodeter Biodegr 64:727–733. 10.1016/j.ibiod.2010.08.006

[CR9] Carrapiço A, Martins MR, Caldeira AT, Mirão J, Dias L (2023) Biosynthesis of metal and metal oxide nanoparticles using microbial cultures: mechanisms, antimicrobial activity and applications to cultural heritage. Microorganisms 11:378. 10.3390/microorganisms11020378 36838343 10.3390/microorganisms11020378 PMC9960935

[CR10] Ciferri O (1999) Microbial degradation of paintings. App Environ Microbiol 65:879–885. 10.1128/AEM.65.3.879-885.1999 10.1128/aem.65.3.879-885.1999 PMC91117 10049836

[CR11] Corbu VM, Gheorghe I, Marinaș IC, Geană EI, Moza MI, Csutak O, Chifiriuc MC (2021) Demonstration of *Allium sativum* extract inhibitory effect on biodeteriogenic microbial strain growth, biofilm development, and enzymatic and organic acid production. Molecules 26:7195. 10.3390/molecules26237195 34885775 10.3390/molecules26237195 PMC8659052

[CR12] Corbu VM, Gheorghe-Barbu I, Marinas IC, Avramescu SM, Pecete I, Geanǎ EI, Chifiriuc MC (2022) Eco-friendly solution based on *Rosmarinus officinalis* hydro-alcoholic extract to prevent biodeterioration of cultural heritage objects and buildings. Int J Mol Sci 23:11463. 10.3390/ijms231911463 36232763 10.3390/ijms231911463 PMC9569761

[CR13] Corbu VM, Gheorghe-Barbu I, Ștefania DA, Vrâncianu CO, Șesan TE (2023) Current insights in fungal importance—a comprehensive review. Microorganisms 11:1384. 10.3390/microorganisms11061384 37374886 10.3390/microorganisms11061384 PMC10304223

[CR14] Di Carlo E, Barresi G, Palla F (2022) Biodeterioration. In: Palla F, Barresi G (eds) Biotechnology and conservation of cultural heritage. Springer International Publishing, Cham, pp 1–30

[CR15] Efenberger-Szmechtyk M, Nowak A, Czyzowska A (2021) Plant extracts rich in polyphenols: antibacterial agents and natural preservatives for meat and meat products. Crit Rev Food Sci Nut 61:149–178. 10.1080/10408398.2020.1722060 10.1080/10408398.2020.1722060 32043360

[CR16] Franco-Castillo I, Hierro L, de la Fuente JM, Seral-Ascaso A, Mitchell SG (2021) Perspectives for antimicrobial nanomaterials in cultural heritage conservation. Chem 7:629–669. 10.1016/j.chempr.2021.01.006

[CR17] Gorbushina AA (2007) Life on the rocks. Environ Microbiol 9:1613–1631. 10.1111/j.1462-2920.2007.01301.x 17564597 10.1111/j.1462-2920.2007.01301.x

[CR18] Greenblatt CL, Baum J, Klein BY, Nachshon S, Koltunov V, Cano RJ (2004) *Micrococcus luteus* - survival in amber. Microb Ecol 48:120–127. 10.1007/s00248-003-2016-5 15164240 10.1007/s00248-003-2016-5

[CR19] Joseph E (2021) Microorganisms in the deterioration and preservation of cultural heritage. Springer Nature, Neuchâtel

[CR20] Kolman K, Nechyporchuk O, Persson M, Holmberg K, Bordes R (2018) Combined nanocellulose/nanosilica approach for multiscale consolidation of painting canvases. ACS Appl Nano Mater 1:2036–2040. 10.1021/acsanm.8b00262

[CR21] Kujović A, Gostinčar C, Kavkler K, Govedić N, Gunde-Cimerman N, Zalar P (2024) Degradation potential of xerophilic and xerotolerant fungi contaminating historic canvas paintings. J Fungi 10:76. 10.3390/jof10010076 10.3390/jof10010076 PMC10817455 38248985

[CR22] Lee C-C (2025) Microbial pigments: fermentative production and biological activities. In: Sharma K (ed) Bio-prospecting of novel microbial bioactive compounds for sustainable development: management of natural resources through microbial conversion into valuable products. Springer Nature, Cham, pp 43–65

[CR23] Mao Z, Qiu H, Shih C, Kang Z (2024) P-13.12: The delta E color dissimilarity analysis of LCD panels SID Symposium Digest of Technical Papers 55:1404-1414 10.1002/sdtp.17382

[CR24] Maťátková O, Michailidu J, Miškovská A, Kolouchová I, Masák J, Čejková A (2022) Antimicrobial properties and applications of metal nanoparticles biosynthesized by green methods. Biotechnol Adv 58:107905. 10.1016/j.biotechadv.2022.107905 35031394 10.1016/j.biotechadv.2022.107905

[CR25] Mendes KF, Sousa RD, Mielke KC (2022) Biodegradation technology of organic and inorganic pollutants. BoD – Books on Demand, London

[CR26] Oriola-Folch M, Campo-Francés G, Nualart-Torroja A, Ruiz-Recasens C, Bautista-Morenilla I (2020) Novel nanomaterials to stabilise the canvas support of paintings assessed from a conservator’s point of view. Herit Sci 8:23. 10.1186/s40494-020-00367-2

[CR27] Pandey P, Kiran U (2020) Degradation of paints and its microbial effect on health and environment. J Crit Rev 7:4879–4884

[CR28] Parulekar-Berde C, Ghoble SS, Salvi SP, Berde VB (2020) Microorganisms and their enzymes as biorestoration agents. In: Yadav AN, Rastegari AA, Gupta VK, Yadav N (eds) Microbial biotechnology approaches to monuments of cultural heritage. Springer, Singapore, pp 71–86

[CR29] Pavić A, Ilić-Tomić T, Pačevski A, Nedeljković T, Vasiljević B, Morić I (2015) Diversity and biodeteriorative potential of bacterial isolates from deteriorated modern combined-technique canvas painting. Int Biodeter Biodegr 97:40–50. 10.1016/j.ibiod.2014.11.012

[CR30] Phulpoto AH, Qazi MA, Mangi S, Ahmed S, Kanhar NA (2016) Biodegradation of oil-based paint by *Bacillus* species monocultures isolated from the paint warehouses. Int J Environ Sci Technol 13:125–134. 10.1007/s13762-015-0851-9

[CR31] Poyatos F, Morales F, Nicholson AW, Giordano A (2018) Physiology of biodeterioration on canvas paintings. J C Physiol 233:2741–2751. 10.1002/jcp.26088 10.1002/jcp.26088 28688195

[CR32] Puvača N, Milenković J, Galonja Coghill T, Bursić V, Petrović A, Tanasković S, Pelić M, Ljubojević Pelić D, Miljković T (2021) Antimicrobial activity of selected essential oils against selected pathogenic bacteria: in vitro study. Antibiotics 10:546. 10.3390/antibiotics10050546 34066788 10.3390/antibiotics10050546 PMC8151751

[CR33] Rubio RF, Bolívar FC (1997) Preliminary study on the biodeterioration of canvas paintings from the seventeenth century by *Microchiroptera*. Int Biodeter Biodegr 40:161–169. 10.1016/S0964-8305(97)00043-7

[CR34] Russo R, Palla F (2023) Plant essential oils as biocides in sustainable strategies for the conservation of cultural heritage. Sustainability 15:8522. 10.3390/su15118522

[CR35] Santos A, Cerrada A, García S, San Andrés M, Abrusci C, Marquina D (2009) Application of molecular techniques to the elucidation of the microbial community structure of antique paintings. Microb Ecol 58:692–702. 10.1007/s00248-009-9564-2 19633806 10.1007/s00248-009-9564-2

[CR36] Savković Ž, Stupar M, Unković N, Knežević A, Vukojević J, Grbić ML (2021) Fungal deterioration of cultural heritage objects. IntechOpen. 10.5772/intechopen.98620

[CR37] Sparacello S, Gallo G, Faddetta T, Megna B, Nicotra G, Bruno B, Giambra B, Palla F (2021) *Thymus vulgaris* essential oil and hydro-alcoholic solutions to counteract wooden artwork microbial colonization. Appl Sci 11:8704. 10.3390/app11188704

[CR38] Spirescu VA, Chircov C, Grumezescu AM, Andronescu E (2021) Polymeric nanoparticles for antimicrobial therapies: an up-to-date overview. Polymers 13:724. 10.3390/polym13050724 33673451 10.3390/polym13050724 PMC7956825

[CR39] Stan D, Enciu A-M, Mateescu AL, Ion AC, Brezeanu AC, Stan D, Tanase C (2021) Natural compounds with antimicrobial and antiviral effect and nanocarriers used for their transportation. Front Pharmacol 12. 10.3389/fphar.2021.723233 10.3389/fphar.2021.723233 PMC8450524 34552489

[CR40] Sterflinger K, Piñar G (2013) Microbial deterioration of cultural heritage and works of art — tilting at windmills? Appl Microbiol Biotechnol 97:9637–9646. 10.1007/s00253-013-5283-1 24100684 10.1007/s00253-013-5283-1 PMC3825568

[CR41] Szczepanowska H (2023) Biodeterioration of cultural heritage: dynamic interfaces between fungi, fungal pigments and paper. MDPI, Basel

[CR42] Triolo PAM (2020) Manuale pratico di documentazione e diagnostica per immagine per i BB.CC. Il Prato, Padova

[CR43] Vergeer M, van den Berg KJ, van Oudheusden S, Stols-Witlox M (2019) Evolon® cr microfibre cloth as a tool for varnish removal. In: van den Berg KJ, Bonaduce I, Burnstock A, Ormsby B, Scharff M, Carlyle L, Heydenreich G, Keune K (eds) Conservation of modern oil paintings. Springer International Publishing, Cham, pp 587–596

