# Supplementary material for: Biodeterioration of canvas paintings: microbial role and development of sustainable treatments for biocontrol
Source: Appl Microbiol Biotechnol. 2025 Aug 13;109(1):181. doi: 10.1007/s00253-025-13553-8 (PMC12350447; doi:10.1007/s00253-025-13553-8)
Supplement: Supplementary file 1 — (DOCX 26.9 MB) [file 253_2025_13553_MOESM1_ESM.docx]

**Supplementary materials**

Journal: **Applied Microbiology and Biotechnology**

**Biodeterioration of canvas paintings: microbial role and development of sustainable treatments for biocontrol**

Giovanna Climaco^1ψ^, Gianmaria Oliva^2ψ*^, Paola Fiore^1*^, Consiglia Tedesco^2^, Stefano Castiglione^2^, Giovanni Vigliotta^2^

^1^ Department of Design and Applied Arts, Conservation School, Fine Arts Academy of Naples, 80138 Naples (NA), Italy

^2^ Department of Chemistry and Biology “A. Zambelli”, University of Salerno, 84084 Fisciano (SA), Italy

^*^ Corresponding author

E-mail address: [gioliva@unisa.it](mailto:gioliva@unisa.it) (G. Oliva); [paola.fiore@abana.it](mailto:paola.fiore@abana.it) (P. Fiore)

^ψ^ Giovanna Climaco and Gianmaria Oliva have contributed equally to the manuscript, and they can be considered as first authors

**Fig. S1** (A) Different purple-red-white spots attributable to microbial activity on relining canvas; (B-C) correspondence of several purple-red-white spots between relining canvas and original canvas.

**Fig. S2** (A) Sample of virgin linen canvas; (B) sample of old relining canvas removed during the conservation of the painting described in this study.

**Fig. S3** Optical microscopy analysis of original canvas and relining canvas samples at magnification 100x and 400x. (A-B) Samples of original canvas TO1 and TO2; (C-D) samples of relining canvas TR1 and TR2.

**Fig. S4** Microscopic observation and morphological analysis of isolated bacteria and fungi.

**Fig. S5** Physical interaction between bacteria and yellow ochre pigment. The experiments were conducted in triplicates. The control represents the BH culture medium with pigment (1.0 g L^-1^) and without bacteria. The red rectangle indicated the formation of aggregates.

**Fig. S6** Physical interaction between bacteria and ivory black pigment. The experiments were conducted in duplicates. The control represents the BH culture medium with pigment (1.0 g L^-1^) and without bacteria. The red rectangle indicated the formation of aggregates.

**Fig. S7** Pigment sequestration in the fungal mycelium. (A) Yellow ochre and (B) ivory black. The experiments were conducted in duplicates. The letter C represents the control.

**Fig. S8** Antimicrobial activity of thymus (T), garlic (G), and rosemary (R) extracts against the isolated bacteria and fungi. For fungi, only rosemary extract has shown antimicrobial activity against the *Penicillum chrysogenum* strains (F2A, F3B, F4B, F6A).
